# Yeast functional screen to identify genetic determinants capable of conferring abiotic stress tolerance in *Jatropha curcas*

**DOI:** 10.1186/1472-6750-10-23

**Published:** 2010-03-20

**Authors:** Nalini Eswaran, Sriram Parameswaran, Balaji Sathram, Bhagyam Anantharaman, Raja Krishna Kumar G, Sudhakar Johnson Tangirala

**Affiliations:** 1 Plant Metabolic Engineering Group, Reliance Life Sciences Pvt Ltd, Dhirubhai Ambani Life Sciences Center, R-282, Thane- Belapur Road, Rabale, Navi Mumbai- 400 701, India; 2 DuPont Knowledge Centre, ICICI Knowledge Park, Genome Valley, Turkapalli, Shamirpet, Hyderabad 500 078, India

## Abstract

**Background:**

Environmentally inflicted stresses such as salinity and drought limit the plant productivity both in natural and agricultural system. Increasing emphasis has been directed to molecular breeding strategies to enhance the intrinsic ability of plant to survive stress conditions. Functional screens in microorganisms with heterologous genes are a rapid, effective and powerful tool to identify stress tolerant genes in plants. *Jatropha curcas *(Physic nut) has been identified as a potential source of biodiesel plant. In order to improve its productivity under stress conditions to benefit commercial plantations, we initiated prospecting of novel genes expressed during stress in *J. curcas *that can be utilized to enhance stress tolerance ability of plant.

**Results:**

To identify genes expressed during salt tolerance, cDNA expression libraries were constructed from salt-stressed roots of *J. curcas*, regulated under the control of the yeast *GAL1 *system. Using a replica based screening, twenty thousand yeast transformants were screened to identify transformants expressing heterologous gene sequences from *J. curcas *with enhanced ability to tolerate stress. From the screen we obtained 32 full length genes from *J. curcas *[GenBank accession numbers FJ489601-FJ489611, FJ619041-FJ619057 and FJ623457-FJ623460] that can confer abiotic stress tolerance. As a part of this screen, we optimized conditions for salt stress in *J. curcas*, defined parameters for salt stress in yeast, as well as isolated three salt hypersensitive yeast strains *shs-2, shs-6 *and *shs-8 *generated through a process of random mutagenesis, and exhibited growth retardation beyond 750 mM NaCl. Further, we demonstrated complementation of the salt sensitive phenotypes in the *shs *mutants, and analyzed the expression patterns for selected *J. curcas *genes obtained from the screen in both leaf and root tissues after salt stress treatments.

**Conclusions:**

The approach described in this report provides a rapid and universal assay system for large scale screening of genes for varied abiotic stress tolerance within a short span of time. Using this screening strategy we could isolate both genes with previously known function in stress tolerance as well as novel sequences with yet unknown function in salt stress tolerance from *J. curcas*. The isolated genes could be over-expressed using plant expression system to generate and evaluate transgenic plants for stress tolerance as well as be used as markers for breeding salt stress tolerance in plants.

## Background

Environmentally inflicted stresses such as extreme temperatures, low water availability, high salt levels, mineral deficiency and toxicity are frequently encountered by plants both in natural and agricultural system that affect plant productivity. According to Bayer [1], abiotic stresses are estimated to reduce yields to less than half of that possible under ideal growing conditions. The efforts to improve crop performance under environmental stresses have been moderately successful as the fundamental mechanisms of stress tolerance in plants are yet to be completely understood. Conventional approaches to breeding crop plants with improved stress tolerance have thus far met with limited success because of the difficulty of breeding tolerance associated traits from diverse plant backgrounds. Hence, an increasing emphasis has been directed to molecular strategies targeted at enhancing the intrinsic ability of the plants to survive stress conditions. Current approaches proposed to date focus attention on identification of genes associated with salinity, drought and abiotic stress resistance, followed by genetic modification of the plants expressing genes enabling them to withstand restrictive growth imposed by unfavourable environmental conditions [2].

Functional screening of microorganisms that express heterologous cDNA libraries is a powerful tool for identifying genes with specific functions, independent of the regulation of their expression [3]. The screening of *E. coli *or yeast expressing plant cDNAs has been used successfully to identify genes that are involved in enhanced stress tolerance [4]. Functional screening of sodium-sensitive yeast expressing a cDNA library of the halotolerant plant sugar beet resulted in identification of the eukaryotic translation initiation factor (eIF1A), and overexpression of the eIF1A has been reported to increase salt tolerance of yeast and *Arabidopsis *[5]. Cadmium (II)-sensitive yeast mutant *ycf1 *has been employed to screen *Arabidopsis *cDNA library to identify cadmium resistance gene, AtPcrs [6]. The genes for allene oxide cyclase and the cytosolic chaperonin containing TCP-1a homologue [4] have been isolated as salt tolerant genes from *B. sexangula *by functional screening on NaCl-containing medium using *E. coli *as the host organism. More recently, Ezawa and Tada [4] reported functional screening in *Agrobacterium *as an effective supplemental method to pre-screen genes involved in abiotic stress tolerance. These results demonstrate that microbial functional screening is an effective tool to quickly identify stress tolerant gene candidates in plants.

Genetic engineering and modern molecular breeding methods have been used to isolate key genes involved in abiotic stress response from characterized model plants such as *Arabidopsis*, and several economically important crops [7,8]. Directly applying these strategies to non-model plants is often challenging. Several other approaches such as DD-RT-PCR, subtractive hybridization, microarray analysis and FOX screening have also led to identification of genes regulated during stress response [2,9,10]. However, a functional effect of the gene(s) identified on stress resistance is not always clearly established from such experimental approaches [11]. While it has been possible in certain instances to extend and utilize the information available on genes regulating and/or conferring salinity, drought and abiotic stress resistance in model plants, the ability to uncover novel or target plant specific genes/pathways regulating abiotic stress tolerance would be limited without a *de novo *screen.

*Jatropha curcas *(Physic nut), a member of Euphorbiaceae family has been identified as a biodiesel plant in tropical countries [12]. Being a Euphorbiaceae member, *J. curcas*, is to a certain extent, drought tolerant and has been documented in certain instances to grow in heavy metal contaminated soils [13]. Commercial plantation of *J. curcas *has been proposed as a sustainable source of biodiesel mainly due to high seed oil content, lipid composition similar to that of fossil diesel and non-competing demand with edible oil supplies [14]. Prospecting of novel genes expressed during stress tolerance from *J. curcas *and utilizing genetic information to modify and enhance the stress tolerance ability of this plant may enable development of *J. curcas *lines capable of producing growth in poor and marginal soils with acceptable oil yields. Besides, these novel genes isolated from *J. curcas *may also be applied for genetic improvement of other agriculturally important crops mediated via heterologous gene expression or to develop saline and drought tolerant varieties.

However, due to limited genome information, large scale gene prospecting to identify genes that are linked to resistance to abiotic stress in *J. curcas *has been challenging. To overcome this limitation, we describe a functional genetic screen that utilizes yeast as a surrogate system to identify and isolate genes involved in stress tolerance. We have used yeast *GAL1 *regulated, cDNA expression libraries derived from *J. curcas*, to develop replica printing based functional screen for identification of yeast transformants expressing heterologous genes that confer abiotic stress tolerance. Through the above screen, we have demonstrated the ability to isolate several novel genes specific to *J. curcas *expressed during abiotic stress tolerance.

## Results and discussion

### Identification of salt stress condition for wild-type yeasts and isolation of salt hypersensitive yeast mutants, *shs-2, shs-6 *and *shs-8*

**Figure 1:**
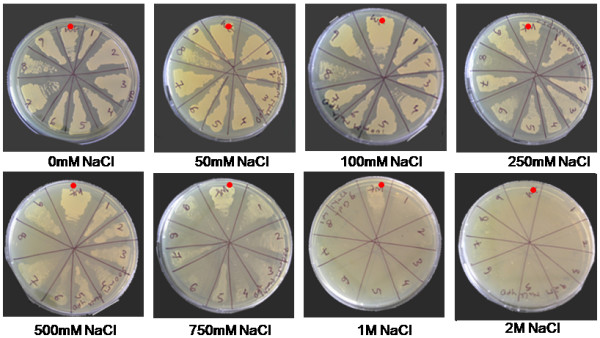
**Identification of salt stress conditions for wild-type *S. cerevisiae *(BY4741)**. Sectors in plates marked with red dot are wild-type, while other sectors represent salt hypersensitive yeast mutants obtained through random UV mutagenesis. Wild-type yeast BY4741 shows salt sensitivity from 500 mM NaCl, with complete growth arrest at 2.0 M NaCl. In contrast, salt hypersensitive mutants *shs-2, shs-4, shs-6 and shs-8 *(isolated in the BY4741 background) show growth retardation from 500 mM. Similar amount of inoculums were used in all the plates.

To identify and isolate *J. curcas *genes involved in salt tolerance, yeast cells were used for functional screening. Yeast cells have been shown to exhibit salt stress, which is reflected as retardation in cell growth. Wild type yeast strains have been reported to show salt sensitivity from 500 mM depending on growth conditions. In our assays we scored for survival of yeast-transformants that could survive at and beyond 750 mM NaCl. This condition selected for the screen at a stringent concentration of 750 mM NaCl, subjected the yeast transformants to ionic as well as osmotic stress, and imposed stringent conditions for salt and drought stresses. Further, to enhance sensitivity of the screen, three salt hypersensitive mutants *shs-2*, *shs-6 *and *shs-8 *were generated by random mutagenesis that exhibited growth retardation beyond 500 mM NaCl (Figure 1). The salt hypersensitive mutant *shs-2 *was used to demonstrate the ability of recovered *J. curcas *genes to complement the salt sensitive defect in this mutant.

**Figure 2:**
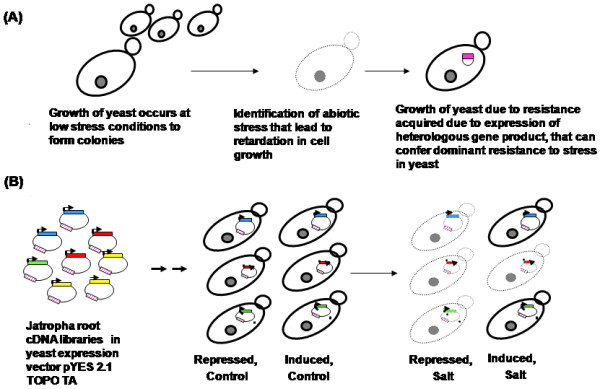
**Schematic illustration of the underlying principle of the functional genetic screen**. Identification and isolation of *J. curcas *genes conferring resistance to abiotic stress in *S. cerevisiae*. In **panel (A) **growth of *S. cerevisiae *colonies under abiotic stress conditions acquired due to heterologous gene expression was represented. In **panel (B) **transformation and screening of *J. curcas *root cDNA library in *S. cerevisiae *for ability to survive stress using inducible promoter was represented.

The underlying basis for the screen is the ability to use a catabolic regulated *GAL1 *promoter [15] to conditionally express a *J. curcas *gene in the yeast cells, cloned into a yeast shuttle vector [16]; while scoring the relative survival of yeast cells exposed to salinity or other abiotic stress to the same yeast transformants, grown under repressed state, subject to similar stress or under control conditions. Scoring the relative growth patterns allows the identification and characterization of the tolerant transformants by plasmid-rescue (Figure 2).

### Construction of cDNA library from *J. curcas *to identify stress responsive genes

To understand the early salt-stress phenotypes and characterize the genes involved in early responses to salt stress on *J. curcas*, 3-4 week old seedlings were treated with various levels of salt stress (see additional file 1: salinity treatment to seedlings). The phenotypic effects of salt stress resulted in observable wilting and drooping in *J. curcas *seedlings. The effect of salt stress was evident at a concentration of 150 mM NaCl, and the effects were pronounced at higher salt concentrations, or when the seedlings were treated for prolonged time (see additional file 1). Further, from this time-course analysis, for salinity stress phenotype in *J. curcas *it was identified that the exposure of roots to 150 mM NaCl for 2 h, at a relative humidity of 40-50% was sufficient to observe early effects of salt stress, which was marked by low level-wilting of young leaf tissue prior to stem drooping (see additional file 1: salinity treatment to seedlings).

Salt and stress pathways in plants are perceived by signalling networks and regulated by transcription machinery [17]. To enrich cDNA libraries for transcripts that represent a greater proportion of early acting factor to later responding regulators, we sampled root tissue at the early-time points of salt stress. *J. curcas *root tissue samples treated at 150 mM, for 2 h time point were used in construction of cDNA libraries using the SMART cDNA synthesis Kit [18] (see Methods).

While plant genomes display large size variations, the number of expressed complement of genes involved in cellular processes are more conserved. Based on the information available for model plant *Arabidopsis *and rice, ~3000-5000 genes out of a transcriptome of ~27000 genes are expressed in root tissue [19,20]. Recent analysis indicates a modest genome size for the *J. curcas*, among Euphorbiaceae members, with an estimated genome size of ~450 Mb that provide an approximation of transcriptome complexity [21]. To adequately represent root transcriptome, titers representing ~48000 c.f.u were recovered in each of the un-amplified pools that largely comprised of full-length cDNA sequences in 45-50% of the genes. This library was used for subsequent functional analysis in yeast.

### Yeast based replica-printing method for isolating *J. curcas *genes involved in abiotic stress

**Figure 3:**
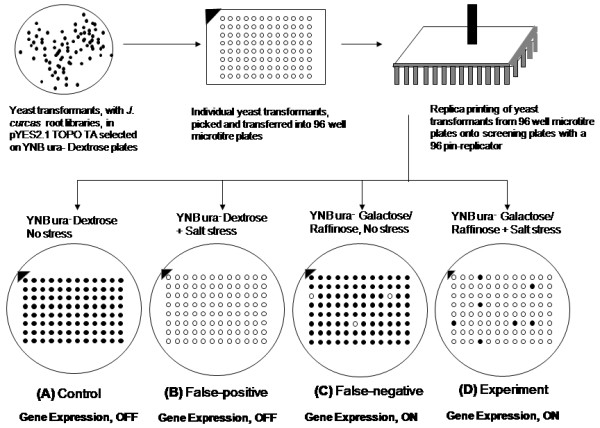
**Replica printing**. Schematic outline of the quadruplet plate based replica-printing to screen yeast transformants to salt stress. In this assay each individual yeast transformant is placed on four selective plates. Comparison of growth of yeast between these plates allows scoring and isolation of transformants expressing genes, conferring stress tolerance.

To increase the probability of representation of each individual clone within these yeast expression libraries and to efficiently transform yeast, we amplified the copy numbers of clones contained in these libraries in *E. coli *(see Methods for details). A total of 20,000 yeast transformants (for the plasmid borne *URA3 *marker) representing the *J. curcas *root cDNA library was screened for salinity tolerance using a replica printing method (Figure 3). The replica printing process was devised to identify and isolate yeast transformants expressing heterologous gene sequences derived from *J. curcas *root cDNA libraries, with a low-rate of false negatives. Screening criteria included scoring relative survival advantage, for each transformant when subject to stress and gene expression status (Figures 2 and 3). This method increased efficiency of gene recovery and reduced the screening errors by providing additional, more exhaustive controls that allowed the identification and elimination of both false-positive as well as false-negative phenotypes. The screening conditions select yeast transformants that are capable of survival under stress (acquired due to expression of *J. curcas *heterologous cDNA) and are able to grow; while eliminating events due to lethality, non-expression and other experimental artefacts.

**Figure 4:**
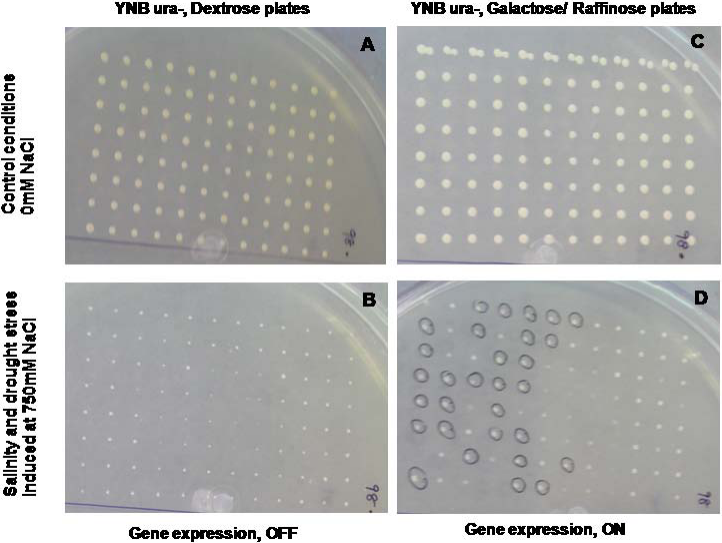
**Yeast transformants expressing genes derived from *J. curcas *root library**. Photographs of representative replica plate screen (as described in Figure 3) demonstrating the ability to isolate yeast transformants during salinity stress. Yeast transformants (selected for plasmid borne *URA3 *marker), expressing genes derived from *J. curcas *root libraries, showing tolerance to stress induced by 750 mM NaCl are marked in **panel D **(black circles). Identical yeast transformants, (compare panel B to D) with repressed gene expression shows arrested growth. **(A) **describes control conditions where gene is repressed and stress is not provided to the yeast cells. Yeast transformants are expected to grow on synthetic selection plates with dextrose as the carbon source. Transformants in type **(B) **conditions (to identify false-positives) are grown in synthetic selection media with dextrose as the carbon source, but treated under stress conditions. In these plates all transformants are expected to show retarded growth, due to the stress conditions. In type **(C) **conditions, yeast transformants are grown on synthetic selection plates containing galactose, but without subjecting them to stress conditions. If the expression of any heterologous gene is detrimental to cell grown, it can be identified, thus eliminating recovery of false-negative transformants in the screen. In type **(D) **conditions, where the gene expression is induced by galactose and the cells are simultaneously subjected to stress, yeast transformants that are capable of survival under stress (acquired due to expression of *J. curcas *heterologous cDNA) are able to grow (Figure 3).

Using the replica-printing based functional screen, 345 candidate yeast transformants expressing cDNA (derived from *J. curcas *roots) were isolated from a pool of 20,000 yeast transformants. A representative data set showing results of phenotypic selection of salt tolerance is presented in (Figure 4). The transformed yeast cells displayed an enhanced ability to tolerate unfavourable conditions imposed by salinity stress at a stringent selection of 750 mM NaCl concentration.

### Plasmid rescue, complementation of salt sensitive yeast phenotypes and sequence analysis

To determine and characterize *J. curcas *cDNA gene sequences that conferred enhanced salinity tolerance at 750 mM NaCl to yeast transformants plasmid rescue was performed as described in Bates [22]. Analysis of the DNA sequence information, recovered from the yeast transformants showed that 32 sequences from 345 clones encoded full-length genes with assignable ORF's derived from *J. curcas; *while the remaining were truncated cDNA sequences. The ability to recover genes from *J. curcas *involved in these processes demonstrates the efficacy of our strategy.

**Figure 5:**
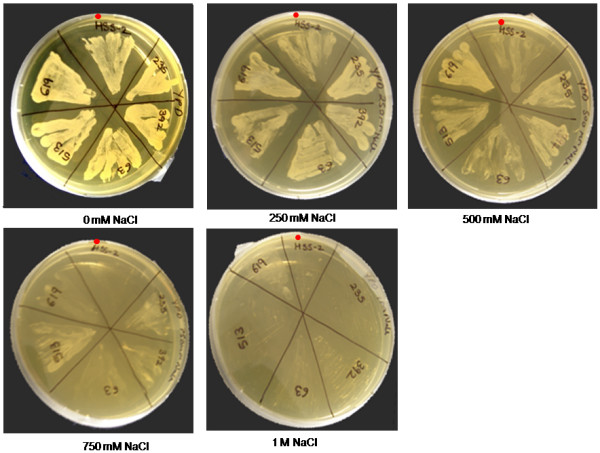
**Functional evaluation of transformants in salt hypersensitive *(shs-2) *mutant**. Plasmid transformation of *shs-2 *with five selected genes. 235, late embryogenesis abundant protein-5; 392, cytosolic ascorbate peroxidase -1; 63, metallothionein; 513, mitochondrial ATP synthase 6 KD subunit; 619, profilin. Single colony of each transformants and mutant strain was plated on YPD media containing range of NaCl concentrations (0 mM, 250 mM, 500 mM, 750 mM and 1 M).

To demonstrate the robustness of the screening method, we have re-transformed the rescued plasmids into wild-type yeast (data not shown) and salt hypersensitive mutant *shs-2 *(Figure 5). We evaluated these transformants for growth at high-salt stress of 750 mM or higher concentration. Several transformants of *shs-2 *mutant allowed growth at 750 mM NaCl. The data demonstrated the ability of the plasmid encoded genes derived from *J. curcas *permitted survival of wild-type yeast transformants (data not shown), and complemented several salt sensitive phenotypes of the *shs-2 *mutants. The data provided functional support for the consistent ability of the recovered genes to confer salt tolerance, and support cell growth at conditions otherwise detrimental to cell survival.

### Computational sequence, annotation and analysis reveals novel abiotic stress tolerant genes from *J. curcas*

Further, sequence analysis and computational annotation reveals presence of novel genes from *J. curcas *involved in abiotic stress tolerance (Table 1). To annotate the sequences, computational searches were performed for similarity to sequences deposited in plant sequence databases (see Methods), that suggest the presence of plant specific regions within these sequences (Table 1). In this analysis 31 out of 32 (~96%) sequences with assignable hit to the sequence databases showed matches greater than the accepted threshold as elaborated by Altshul *et al*. [23] to other known plant genes; while for a single sequence, significant matches to existing sequences in the sequence database was not apparent (Table 1). This sequence could represent novel determinant of salinity and drought stress tolerance, whose role needs to be further established. Of the 31 sequences, 7 sequences showed match to encoded ribosomal proteins, while 1 sequence was annotated as a eukaryotic translation initiation factor SUI1. Twenty three sequences have been reported to have a strong role in conferring abiotic stress tolerance in other plant species (Table 1).

**Table 1:** Annotation of 32 full length genes, derived from *J. curcas*, obtained from yeast functional genetic screen tolerant to abiotic stress*

**Sr. No**.	Clone ID	**GenBank Accession No**.	Putative function of gene	Length of gene (bp)	References*
1.	JcRHDYT30	FJ489601	Allene oxide cyclase	777	Yamada *et al*. 2002 [28]

2.	JcRHDYT70	FJ489602	Thioredoxin H-type (*TRX-h*)	357	Hong *et al*. 2004 [25]; Serrato *et al*. 2004 [26]

3.	JcRHDYT63	FJ489603	Metallothionein	234	Jin *et al*. 2006 [24]

4.	JcRHDYT49	FJ489604	Heterotrophic ferredoxin	492	Sheokand and Brewin, 2003 [41]

5.	JcRHDYT91	FJ489605	Defensin	234	Zhang *et al*. 2008 [42]

6.	JcRHDYT95	FJ489606	Calmodulin-7 (*CAM-7*)	810	Pardo *et al*. 1998 [43]

7.	JcRHDYT29	FJ489607	Major allergen Pru ar1-like protein	495	Mbeguie *et al*. 1997 [30]

8.	JcRHDYT19	FJ489608	S18.A ribosomal protein	495	Williams *et al*. 2003 [27]

9.	JcRHDYT69	FJ489609	60S ribosomal protein L18a	537	Carroll *et al*. 2008 [44]

10.	JcRHDYT108	FJ489610	Protease inhibitor/seed storage/lipid transfer protein family	348	Choi *et al*. 2008 [45]

11.	JcRHDYT174	FJ489611	Unknown protein	195	

12.	JcRHDYT231	FJ619041	Membrane protein -2	189	

13.	JcRHDYT235	FJ619042	Late embryogenesis abundant protein 5 (*LEA-5*)	267	Hundertmark and Hincha, 2008 [29]

14.	JcRHDYT365	FJ619043	Cold-induced plasma membrane protein	174	Imai *et al*. 2005 [46]

15.	JcRHDYT392	FJ619044	Cytosolic ascorbate peroxidase -1 (*Apx-1*)	753	Badawi *et al*. 2004 [31]

16.	JcRHDYT619	FJ619045	Profilin-like protein	384	Ramachandran *et al*. 2000 [33]

17.	JcRHDYT618	FJ619046	Caffeoyl-CoA-O-methyltransferase (*CCoAOMT*)	741	Day *et al*. 2009 [47]

18.	JcRHDYT557	FJ619047	Eukaryotic translation initiation factor SUI1	381	Langland *et al*. 1996 [48]

19.	JcRHDYT401	FJ619048	Copper chaperone	282	Wintz and Vulpe, 2002 [32]

20.	JcRHDYT391	FJ619049	Ubiquitin conjugating enzyme 2 (*JcE2*)	447	Criqui *et al*. 2002 [49]

21.	JcRHDYT513	FJ619050	Mitochondrial ATP synthase 6 KD subunit (*JcMtATP6*)	171	Zhang *et al*. 2006 [17]

22.	JcRHDYT442	FJ619051	Ferritin-2, chloroplast precursor	771	Masuda *et al*. 2001 [50]

23.	JcRHDYT483	FJ619052	Annexin-like protein	945	Gidrol *et al*. 1996 [51]

24.	JcRHDYTC-4	FJ619053	Al-induced protein	711	Taylor *et al*. 1997 [52]

25.	JcRHDYT399	FJ619054	Avr9/cf-9 rapidly elicited (*JcACRE*) gene	231	Rowland *et al*. 2005 [53]

26.	JcRHDYT354	FJ619055	60S ribosomal protein L39	156	Carroll *et al*. 2008 [44]

27.	JcRHDYT370	FJ619056	Ribosomal protein L37	291	Carroll *et al*. 2008 [44]

28.	JcRHDYT381	FJ619057	Ribosomal protein L15	729	Carroll *et al*. 2008 [44]

29.	JcRHDYT413	FJ623457	40S ribosomal protein S15	456	Carroll *et al*. 2008 [44]

30.	JcRHDYT461	FJ623458	40S ribosomal S18	459	Van Lijsebetkens *et al*. 1994 [54]

31.	JcRHDYT512	FJ623459	Plant lipid transfer/seed storage/trypsin-alpha amylase inhibitor	306	Lin *et al*. 2005 [55]

32.	JcRHDYT591	FJ623460	Low-molecular weight cysteine rich 69	234	Vanoosthuyse *et al*. 2001 [56]

*Sequence ortholog with previous reports to abiotic stress tolerance have been marked with references in the table.

To understand the possible role of the genes and regulatory pathway for abiotic stress tolerance in *J. curcas*, we assigned functional classes for the gene sequences recovered in the present study. Comparison of functional annotation for several of the sequences obtained from plasmid rescue indicated a possible role in tolerance to abiotic stress. Some of the sequence orthologs of these genes have been reported to be involved in conferring stress tolerance in other plant species. We find among the list of known functions sequence classes mechanistically involved in abiotic stress tolerance due to wounding, response to cellular redox or oxidative damage, and modulation of cell physiology in response to stresses. For example, previous investigations indicate the involvement of metallothioneins [24], thioredoxins, cellular redox machinery [25,26] and factors involved in the regulation of protein translation [27] to be associated with stress resistance in rice and *Arabidopsis*. Among gene sequences listed in Table 1, the most notable examples with implied roles in stress tolerance are discussed below: we isolated a sequence ortholog corresponding to allene oxide cyclase. Sequences of allene oxide cyclase, isolated from mangrove have been reported to confer salinity stress tolerance in multiple organisms [28]. Previous research data also indicate that accumulation of late embryogenesis abundant protein (*LEA-5*) transcripts in response to stress conditions such as cold, drought, UV light, salinity and wounding [29]. In this report, we isolated *J. curcas LEA-5 *like sequences, suggesting a conservation in stress response pathway. Additionally, we identified genes encoding major allergen *Pru ar1*-like proteins, defensin and protease inhibitor/seed storage/lipid transfer protein family reported to get up-regulated upon pathogen attack and/or environmental stimuli [30]. Plants have been known to have specific mechanism for detoxification of reactive oxygen species (ROS) induced in stress response which includes activation expression of antioxidant enzymes such as cytosolic ascorbate peroxidase-1 (*Apx1*). Experiments involving over-expression of *Apx-1 *in transgenic tobacco, conferred enhanced tolerance to salt and water stress, by effectively detoxifying accumulated H_2_O_2 _in the chloroplasts [31]. Mitochondrial ATP synthase 6 KD subunit (*MtATP6*) is reported to be involved in NaHCO_3 _induced stress, whose over-expression in tobacco led to greater salt stress tolerance [17]. Al-induced protein and copper chaperone have been implicated to provide protection to cellular function during cytotoxic reactions caused by metal ions [32]. We identified *J. curcas *sequence ortholog of a profilin. Profilins have previously been implicated in root elongation and root hair system, a response that is directly linked to response of plants to adapt and source water during drought resistance [33]. The high frequency of occurrence of functionally conserved genetic components indicates a possible conservation in some of the genetic pathways and/or the genes regulating salinity and drought stress among diverse plants [2]. The fact that some of these factors are conserved suggests that certain aspects of mechanism of salt and water deficit stress sensing and response may be evolutionarily conserved among diverse taxa.

### Gene-expression analysis of selected *J. curcas *sequences involved in salinity stress tolerance using semi-quantitative RT-PCR

**Figure 6:**
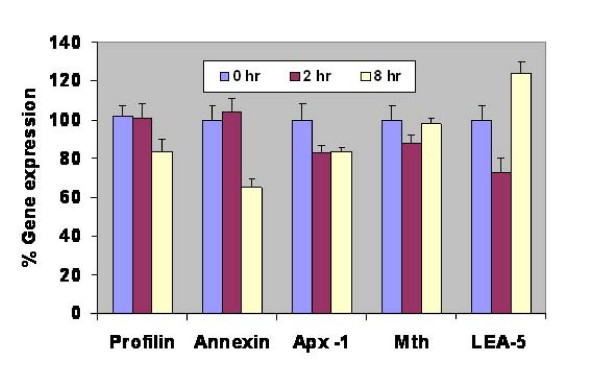
**Semi-quantitative RT-PCR analysis of expression of five different genes in leaf tissue after treating *J. curcas *seedlings with 150 mM NaCl**. *Apx-1*, cytosolic ascorbate peroxidase -1; *Mth*, metallothionein; *LEA-5*, late embryogenesis abundant protein-5. Bars represent S.E. of mean (*n *value is 3).

**Figure 7:**
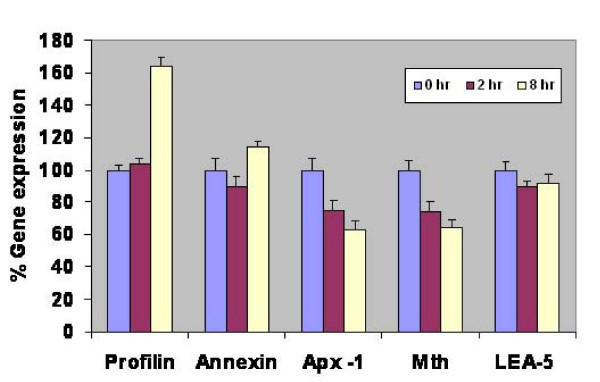
**Semi-quantitative RT-PCR analysis of expression of five different genes in root tissue after treating *J. curcas *seedlings with 150 mM NaCl**. *Apx-1*, cytosolic ascorbate peroxidase -1; *Mth*, metallothionein; *LEA-5*, late embryogenesis abundant protein-5. Bars represent S.E. of mean (*n *value is 3).

To understand and correlate the gene expression pattern of selected genes to functional roles in stress determination, we performed gene expression analysis in leaf and root tissues of *J. curcas*. In the present study, we selected five genes i.e., late embryogenesis abundant protein 5 (*LEA-5*), cytosolic ascorbate peroxidase (*Apx-1*), metallothionein, profilin and annexin, based on the diverse functional classes and varied mechanisms implicated in stress avoidance/tolerance pathways in model plants. While our functional screen has been designed to identify functional genes to abiotic stress tolerance, rather than rely on changes in gene expression; several reports have correlated changes in gene expression to possible gene functions mediating stress tolerance [8]. To correlate gene function and gene-expression status for each of the genes, semi-quantitative RT-PCR analysis was conducted. We assayed for changes in transcript abundance for each of the genes that have been normalized to actin, in both leaf and root tissue, that were either control treated or salt stressed, before sampling at various time-intervals (see Methods). Changes in expression for each transcript normalized to actin have been plotted in Figure 6 and Figure 7.

Analysis of the gene-expression data suggests dynamic changes in the transcript abundance of these genes, with changes in transcript level being apparent from 2 h time-point, indicating an early regulation of the genes in response to salt stress. Gene expression of the normalized transcripts suggests both up-regulation as well as repression of the transcripts. We noted increased expression of profilin in root tissue between 2 h to 8 h time-points of exposure to 150 mM salt stress; whereas profilin was repressed in leaves (Figure 7 vs. Figure 6) consistent with previously reported function during salt stress [33]. In contrast, we observed an initial repression in the expression of annexin that was subsequently induced in the root-tissue, while the expression was down-regulated upon salt-stress in the leaves (Figure 6 vs. Figure 7). Transcript abundance in the levels of *Apx-1 *and metallothioneins were down-regulated in roots, while a more dynamic expression pattern was observed in the leaf tissues, implicating a role in stress adaptive responses [31]. Among the transcripts we profiled in this study, only *LEA-5 *was up-regulated in leaves at the 8 h time interval, after initial down-regulation at the 2 h time-point; while in roots *LEA-5 *expression was unaffected by salt stress. Such an induction of *LEA-5 *in leaf-tissue demonstrated either a longer range signal or the terminal effects of long exposure to ions by the roots. Further experiments are required to de-lineate these effects. The gene-expression data, indirectly suggests varied modes of gene-regulation between the leaf and root tissue (Compare Figure 6 to Figure 7). The data indicates a complex framework for gene regulation during adaptation to salt stress in different tissues. The data in conjunction with gene function may help unravel certain aspects of plant abiotic stress response.

## Conclusion

The approach described in the report provides a rapid and universal assay system for large scale screening of genes for varied abiotic stress tolerance within a short span of time. Using the above screening strategy, we could isolate genes with previously known function in stress tolerance and novel sequences with unknown function in salt stress tolerance from *J. curcas*. The semi-quantitative RT-PCR expression analysis of selected genes revealed differential expression in leaves and roots in response to salt stress. The isolated genes could be over-expressed using plant expression system to generate and evaluate transgenic plants for stress tolerance as well as be used as markers for breeding salt stress tolerance in plants. With minor modification, the functional screening methodology reported in the present study may be extended to isolate plant genes that confer tolerance to a diverse array of possible abiotic stresses. The stress conditions that can be screened with this assay include a) pH stress (due to acidic or basic conditions) b) oxidative stresses c) unfavorable temperature d) heavy metal and e) DNA damage/radiation (by exposure to UV, chemicals that induce DNA breakage).

## Methods

### Plant material

Seeds of *J. curcas *(S003) were collected from Reliance Life Sciences' agricultural farm at Kakinada, South of Andhra Pradesh, India. Seed coats were removed from the seeds, surface sterilized with 70% ethanol followed by 5% hypochloride solution prior to being placed onto MS basal salts [34] distributed into 150-mm culture tubes. MS basal salts supplemented with 3% sucrose and solidified with 0.8% agar (Hi-Media, India) were used to grow seeds. The cultures were maintained at 23-25°C at 50-60% RH, under long day conditions as described previously [35].

### Stress treatment and sample collection

Young 3-4 week-old *in vitro *germinated *J. curcas *seedlings were removed from the media and randomly separated into groups of 15-20 plantlets. Roots (both main and lateral roots) of these plants were either placed in sterile water or into 150 mM NaCl solution for 2 h. The treated *Jatropha *root tissue from plants that were untreated or treated were dissected and frozen in liquid nitrogen, prior to being stored at -80°C.

### Total RNA isolation

*J. curcas *root samples control treated or challenged with 150 mM NaCl were used to generate pools of cDNA. Total RNA for cDNA synthesis was extracted from *J. curcas *root samples using Qiagen RNA Miniprep Kit (Qiagen, Germany). Briefly, the root tissue samples were homogenized to a fine powder in liquid nitrogen and total RNA was extracted as described in the Plant mini RNA prep kit (Qiagen, Germany). Total RNA was estimated spectrophotometrically using Nanodrop.

### Construction of cDNA and expression libraries

**Figure 8:**
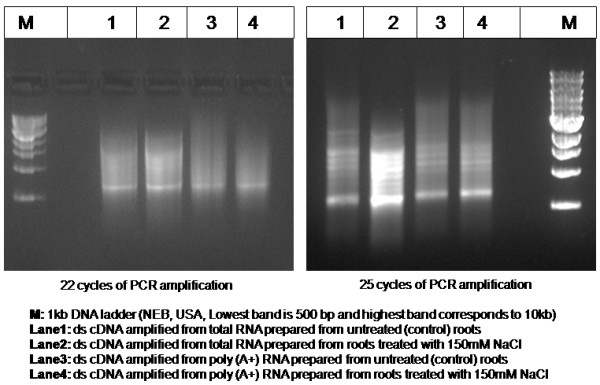
**Amplicons obtained from *J. curcas *L. root tissue**. Gel analysis of amplicons after amplification of double-stranded cDNA library pools as described in the SUPER SMART cDNA construction kit. Yield and distribution of cDNA sizes after 22 or 25 cycles of PCR amplification (as described in Table 2), for double-stranded cDNA prepared from various root RNA pools (marked in the legend) have been shown. Amplicon size distributions ranged from 0.5 to >5.0 kb.

To prepare poly (A+) mRNA pools suitable for cDNA synthesis, 10 μg total RNA was treated with RNAase free DNAaseI (Sigma-Aldrich, St Louis, USA) for 15-20 min at 37°C, and mRNA fraction was enriched using oligo-d(T) beads (Oligotex, Qiagen, Germany). First strand cDNA pools were synthesized from normalized amounts of RNA either derived from untreated root tissue or from tissue challenged with salt stress, using PowerScript reverse transcriptase (Takara, CloneTech) as described in the Super SMART cDNA synthesis Kit [18]. Double stranded DNA was generated through PCR amplification using conditions described in Table 2[18]. The cDNA pools were size separated using NucleoSpin columns (BD CloneTech, USA). The yield and quality of the amplicons were monitored on agarose gel analysis. A profile of amplification patterns obtained after 22 and 25 cycles of PCR amplifications as described in the Super SMART cDNA synthesis Kit [18,36] is shown in Figure 8. A schematic diagram elaborating the construction of *Jatropha *root cDNA libraries is outlined (see additional file 2:-process flow).

**Table 2:** PCR cycling conditions and primer information used for double stranded cDNA generation and colony PCR analysis, as described in SMART cDNA Library Construction Kit [18].

Process	Cycling Conditions	Primer information	
**cDNA synthesis**	95°C - 2 min ↓ 95°C - 30 sec 65°C - 30 sec 68°C - 4 min repeated 18-24 cycles ↓ 68°C - 15 min Hold at 4°C	**SMART IV™ Oligonucleotide (10 μM)** 5'-AAGCAGTGGTATCAACGCAGAGTGGCCATTACGGCCGGG-3' **CDS III/3' PCR Primer (10 μM)** 5'-ATTCTAGAGGCCGAGGCGGCCGACATG-d(T)_30_N-1N-3' **5' PCR Primer (10 μM)** 5'AAGCAGTGGTATCAACG CAGAGT-3'	

**Colony PCR**	95°C - 5 min ↓ 95°C - 30 sec 55°C - 30 sec 72°C - 3 min repeated 29 cycles ↓ 72°C - 5 min Hold at 4°C	**Forward Primer: *Gal 1*** 5'-AATATACCTCTATACTTTAACGTC-3' **Reverse Primer: V5/6XHIS** 5'-ACCGAGGAGAGGGTTAGGGAT-3'	

### Screening for saline tolerant yeast strain and isolation of *shs *mutants

To identify and define the condition that lead to salt stress to wild-type yeast BY4741 (See additional file 3: genotype details) (yeast strain obtained from EUROSCARF, Germany), we measured relative growth of yeast (YPD media 2% Peptone, 1% Yeast extract, 2% Dextrose, solidified with 1.5% Agar, Hi-Media, Mumbai, India) under a range of salinity stress, from 0.0 mM NaCl to 2.0 M NaCl. Salt-hyper-sensitive mutants were isolated through UV induced random mutagenesis and selected on salt containing plates by replica-plating.

### Yeast functional screening

To express the cDNA libraries in yeast, the cDNA library amplicons were cloned into a yeast expression vector, pYES 2.1 TOPO TA (Invitrogen, Carlsbad, USA). The pYES 2.1 TOPO TA is a *E. coli*-*Yeast *shuttle vector [16], propagated in *E. coli *using bacterial selection marker for Amp^r^; while the transformants in the yeast BY4741 strain background are selected for the *URA3 *marker. Cloning of gene of interest downstream of the *GAL1 *promoter allows regulated gene expression of the library in yeast (Invitrogen, Carlsbad, USA). The *J. curcas *root cDNA libraries in pYES 2.1 TOPO TA were transformed into *E. coli *TOP 10F' (Invitrogen, Carlsbad, USA). Subsequently, the cDNA library were recovered from the plates and plasmid DNA representing the pooled libraries, derived from *J. curcas *root cDNA was extracted using Plasmid Midi preparation Kit (Qiagen, Germany).

### Plasmid transformation of yeast

Plasmid transformation of yeast (*Saccharomyces cerevisiae*) was accomplished using PEG-lithium acetate based transformation protocols [37], while the plasmid selection in yeast was based on the *URA3 *marker borne on the yeast expression plasmid pYES2.1 TOPO TA (Invitrogen, Carlsbad, USA). Amplified plasmids containing cloned inserts derived from *J. curcas*, regulated by the galactose-inducible *GAL1 *promoter, were transformed into wild-type yeast strain BY4741; following heat-shock at 15 min at 42°C, the yeast cells were revived in YPD media and plated on synthetic minimal medium plates lacking uracil (see additional file 4: stock composition), and placed at 23-25°C for 48-96 h.

### Replica printing

The yeast (BY4741) containing *J. curcas *root cDNA library clones, in *GAL1 *regulated yeast expression systemwere screened for salinity and drought resistance. Twenty thousand yeast transformants were picked and inoculated into sterile 96 well U-bottom microtitre plates (Nunc, USA) containing synthetic selection media (see additional file 5: composition of synthetic selection media). Subsequently, the individual yeast transformants were replica printed on quadruplet selection plates containing either 0 mM NaCl or 750 mM NaCl using a 96-pin replicator (Nunc, USA)(Figure 3). Under these screening conditions each individual yeast transformant (arrayed from 96-well plates) was tested for its ability to grow under four conditions described earlier. The *J. curcas *cDNA expression in yeast could be regulated by the yeast *GAL1 *system [38].

### Back transformation to *E. coli*

Isolated yeast transformants were grown on synthetic selection media containing 2% dextrose without salt (see additional file 5: composition of synthetic selection media) for 36-72 h. The transformants were lysed with 10 U/μl lyticase (Sigma-Aldrich, St Louis, USA) and 2-4% SDS. Nucleic acid fraction recovered from yeast were purified with two sequential rounds of phenol:chloroform:isoamyl alcohol (25:24:1) extraction, followed by ethanol precipitation of nucleic acids. To analyze the inserts cloned in the pYES2.1 TOPO TA based yeast expression plasmid, nucleic acid preparations recovered from individual yeast transformants showing resistance to salinity stress were backtransformed into *E. coli *via electroporation (GenePulser II, BioRad, USA) [39].

### Colony PCR and Plasmid DNA preparation

*E. coli *backtransformants for yeast expression plasmids were analyzed for inserts by colony PCR analysis with the *GAL1 *and *V5/6XHIS *primers as described in the pYES2.1 TOPO TA kit (Invitrogen, Carlsbad, USA). The conditions for colony PCR analysis were provided in Table 2. *E. coli *re-transformants (representing the yeast plasmid expression vector containing *Jatropha *cDNA) were grown and plasmid DNA extracted as described in Qiagen plasmid miniprep kit (Qiagen, Germany).

### Sequencing

Sequencing of *Jatropha *cDNA conferring salinity tolerance was performed as described with BigDye Sequencing Kit (ABI, USA), and analyzed on Genetic Analyzer (ABI 3100, USA). Sequencing reaction was with vector specific *GAL1 *and *V5/6XHIS *primers

### Computer analyses and annotation

Prior to annotation, the sequences were subjected to quality check and vector masking using NCBI's UniVec http://www.ncbi.nlm.nih.gov/blast/. To understand and assign functional classes to the sequence information determined in this screen, we performed computational searches against sequence databases at NCBI http://www.ncbi.nlm.nih.gov/blast/ and TIGR plant transcript assemblies http://tigrblast.tigr.org/euk-blast/plantta_blast.cgi using the BLAST algorithm [23,40].

### Evaluation of transformants in hyper-salt sensitive mutant

Plasmid transformation of yeast hyper-salt sensitive mutant (*shs-2*) with five selected genes viz., *LEA 5*, mitochondrial ATP synthase 6 KD subunit, cytosolic ascorbate peroxidase, metallothionein and profilin was done as mentioned earlier. Single colony of each transformants and mutant strain was dissolved in 100 μl sterile water. YPD media containing range of NaCl concentrations (0 mM, 250 mM, 500 mM, 750 mM and 1 M) were prepared in Petri dishes. Each Petri plate was divided into six sectors. 10 μl of each yeast transformants and the mutant strain were patched on the salt series plates uniformly and incubated overnight at 30°C incubator. Survival of transformants was scored against hyper-salt sensitive mutant strain.

### Semi-quantitative RT-PCR expression analysis of selected genes

The semi-quantitative RT-PCR expression of selected genes was performed with specific oligonucleotide primers (Table 3) on first strand cDNA synthesised from RNA isolated from root and leaf tissues samples of 21-day-old *J. curcas *seedlings. The total RNA was extracted from the 150 mM salt treated leaves and roots at different durations of 0 h, 2 h and 8 h as described in the Plant mini RNA prep kit (Qiagen, Germany). The total RNA was estimated spectrophotometrically at 230, 260 and 280 nm (Nanodrop). Eight hundred ng of total RNA was taken to synthesize the first strand cDNA with oligodT using Superscript reverse transcriptase (Invitrogen) as per manufacturer's protocol. For PCR, 1 μL of cDNA was used as DNA template in a reaction volume of 50 μL using PCR master mix (Lucigene) with cycling conditions of 95°C for 5 min, 95°C for 1 min, 60°C for 1 min, 72°C for 1 min. The amplification reaction was carried out for 32 cycles for all genes with a final extension at 72°C for 10 min. *J. curcas *actin gene was used as an internal control for analysis of gene expression. RT-PCR analyses were carried out with three independent total RNA samples.

**Table 3:** List of forward and reverse primers used for semi-quantitative RT-PCR.

Name of Gene*	Forward primer	Reverse primer
*LEA-5*	5' ATGGCTCGCCCTTTCTCAAACG 3'	5' TCAGTTTTTCTTCAGAAGCATAGCTCTTAATTCGG 3'

*Apx-1*	5'ATGGCTAAGAACTATCCAAAAGTAAGC GAAGAGTA3'	5' TTAGGCATCAGCAAATCCCAGCTCTG3'

Metallothionein	5' ATGTCTTGCTGCGGAGGAAACTG 3'	5' CACACGACAGGGTTTGAGAAGGTAC 3'

Profilin	5' ATGTCGTGGCAAACATACGTAGATGAGC 3'	5' CCAAGCCTTTCGACAATCATGTTGC 3'

Annexin	5' ATGGCTACCATTGTTGTTCCTGCC 3'	5' AGCTGTGTCTTGCTCCTTGTAGTGAG 3'

*LEA-5, late embryogenesis abundant protein-5; *Apx-1*, cytosolic ascorbate peroxidase -1.

Quantitation of RT-PCR product was determined by densitometer. Two μl of RT-PCR products derived from target gene and actin gene were resolved on 2%agarose gels stained with ethidiumbromide. Densitometeric scan analysis was carried out using Kodak MI Imaging software program as per the supplier's instructions. Percent gene expression was determined by normalizing values against internal control actin. Values were represented as per cent of gene expression with respect to corresponding controls and plotted using Microsoft Excel.

### Accession Numbers

Sequence data from this article can be found in the GenBank data libraries under accession numbers FJ489601-FJ489611, FJ619041-FJ619057 and FJ623457-FJ623460.

## Authors' contributions

NE carried out the functional screen studies, sequence analysis and helped in drafting the figures and tables. BS assisted in the functional screen and sequence analysis. BA assisted in the functional screen. SP conceived the study, participated in its design and helped in drafting the manuscript. RKK carried out semi-quantitative RT-PCR expression analysis along with NE. TSJ coordinated the whole project and drafted the manuscript. All authors read and approved the final manuscript.

## Supplementary Material

Additional file 1 **Salinity treatment to seedlings**. Twenty one-day-old *J. curcas *seedlings were treated with 0, 150 mM, 250 mM and 750 mM NaCl for 0 h, 2 h, 4 h and 8 h. Total RNA was extracted from root and leaf tissues of 150 mM NaCl treated seedlings at 0 h, 2 h and 8 h and semi- quantitative RT-PCR expression of selected *J. curcas *genes was performed. Click here for file

Additional file 2 **Process flow**. Process flow of genetic screen: Outline of the process to identify and isolate specific genes from *J. curcas *involved in abiotic stress responses using yeast functional genetic screen. Click here for file

Additional file 3 **Genotype details**. Strain details of genotype of yeast, *Saccharomyces cerevisiae *used in the screen. Click here for file

Additional file 4 **Stock composition**. Stock compositions used in synthetic media Click here for file

Additional file 5 **Composition of synthetic selection media used in the functional screen**. Composition of synthetic selection media used in the functional screen Click here for file
