# Supplementary figures and images for: Yeast functional screen to identify genetic determinants capable of conferring abiotic stress tolerance in Jatropha curcas
Source: BMC Biotechnol. 2010 Mar 20;10:23. doi: 10.1186/1472-6750-10-23 (PMC2851662; doi:10.1186/1472-6750-10-23)

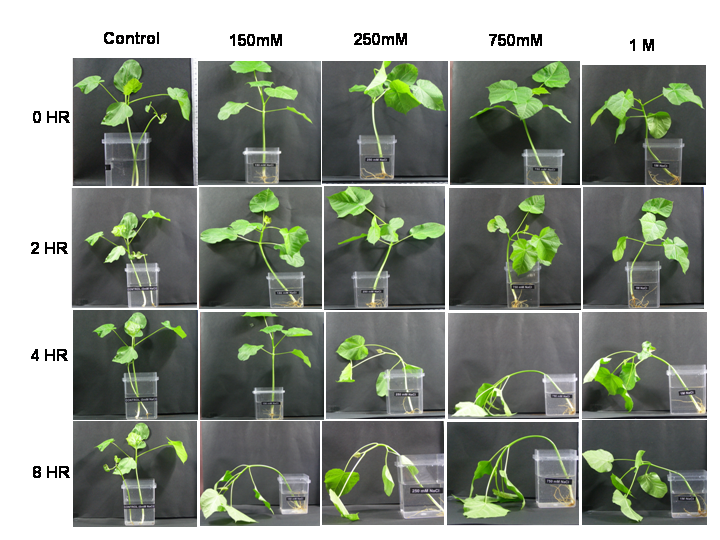

Supplement: Additional file 1 — Salinity treatment to seedlings. Twenty one-day-old J. curcas seedlings were treated with 0, 150 mM, 250 mM and 750 mM NaCl for 0 h, 2 h, 4 h and 8 h. Total RNA was extracted from root and leaf tissues of 150 mM NaCl treated seedlings at 0 h, 2 h and 8 h and semi- quantitative RT-PCR expression of selected J. curcas genes was performed. [file 1472-6750-10-23-S1.TIFF]
