# Supplementary material for: Yeast functional screen to identify genetic determinants capable of conferring abiotic stress tolerance in Jatropha curcas
Source: BMC Biotechnol. 2010 Mar 20;10:23. doi: 10.1186/1472-6750-10-23 (PMC2851662; doi:10.1186/1472-6750-10-23)
Supplement: Additional file 2 — Process flow. Process flow of genetic screen: Outline of the process to identify and isolate specific genes from J. curcas involved in abiotic stress responses using yeast functional genetic screen. [file 1472-6750-10-23-S2.PPT]

## Slide 1
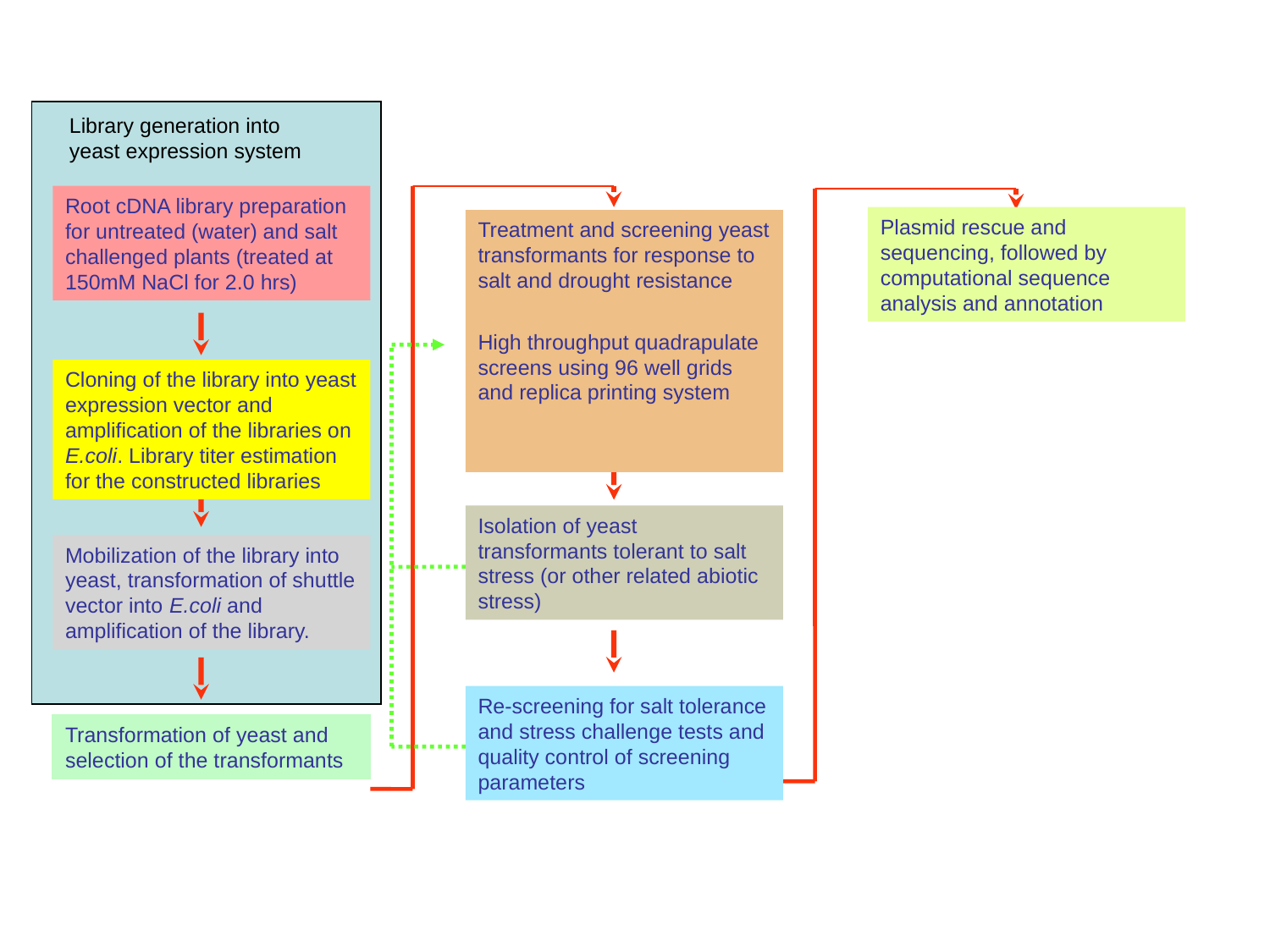

Library generation into yeast expression system
Root cDNA library preparation for untreated (water) and salt challenged plants (treated at 150mM NaCl for 2.0 hrs)
Transformation of yeast and selection of the transformants
Re-screening for salt tolerance and stress challenge tests and quality control of screening parameters
Plasmid rescue and sequencing, followed by computational sequence analysis and annotation
Treatment and screening yeast transformants for response to salt and drought resistance
High throughput quadrapulate screens using 96 well grids and replica printing system
Cloning of the library into yeast expression vector and amplification of the libraries on E.coli. Library titer estimation for the constructed libraries
Isolation of yeast transformants tolerant to salt stress (or other related abiotic stress)
Mobilization of the library into yeast, transformation of shuttle vector into E.coli and amplification of the library.
