# Supplementary material for: Yeast functional screen to identify genetic determinants capable of conferring abiotic stress tolerance in Jatropha curcas
Source: BMC Biotechnol. 2010 Mar 20;10:23. doi: 10.1186/1472-6750-10-23 (PMC2851662; doi:10.1186/1472-6750-10-23)
Supplement: Additional file 3 — Genotype details. Strain details of genotype of yeast, Saccharomyces cerevisiae used in the screen. [file 1472-6750-10-23-S3.DOC]

**Additional file 3:** Strain details of genotype of yeast, *Saccharomyces cerevisiae* used in the screen.

| **Organism** | **Accession Number** | **Strain** | **Genotype** |
| --- | --- | --- | --- |
| *Saccharomyces cerevisiae* | Y00000 | BY4741 | *MATa; his3Δ1; leu2Δ0; met15Δ0; ura3Δ0* |
