# Supplementary material for: Yeast functional screen to identify genetic determinants capable of conferring abiotic stress tolerance in Jatropha curcas
Source: BMC Biotechnol. 2010 Mar 20;10:23. doi: 10.1186/1472-6750-10-23 (PMC2851662; doi:10.1186/1472-6750-10-23)
Supplement: Additional file 4 — Stock composition. Stock compositions used in synthetic media [file 1472-6750-10-23-S4.DOC]

**Additional file 4:** **Stock compositions used in synthetic media**

| **Dropout (10X)** | **Arginine**  **Isoleucine**  **Phenylalanine**  **Threonine**  **Valine**  **Adenine**  **Tryptophane**  **Aspartic acid**  **Proline**  **Serine** | **200mg/L**  **500mg**  **500mg**  **2000mg**  **1500mg**  **2000mg**  **2000mg**  **500mg**  **500mg**  **500mg** |
| --- | --- | --- |
| **YNB + Tyrosine (10X)** | Yeast Nitrogen Base  Tyrosine | 6.7gm/100ml  30mg |
| **Histidine (100X)** | Histidine | 50mg/10ml |
| **Methoinine (100X)** | Methoinine | 50mg/10ml |
| **Leucine (100X)** | Leucine | 100mg/10ml |
| **Lysine (100X)** | Lysine | 100mg/10ml |
