# Supplementary material for: Yeast functional screen to identify genetic determinants capable of conferring abiotic stress tolerance in Jatropha curcas
Source: BMC Biotechnol. 2010 Mar 20;10:23. doi: 10.1186/1472-6750-10-23 (PMC2851662; doi:10.1186/1472-6750-10-23)
Supplement: Additional file 5 — Composition of synthetic selection media used in the functional screen. Composition of synthetic selection media used in the functional screen [file 1472-6750-10-23-S5.DOC]

**Additional file 5.** Composition of synthetic selection media used in the functional screen

|  | Components | Amount in ml/L |
| --- | --- | --- |
| **YNB URA^-^  DEXTROSE**  **(Control)*** | Dropout media  Histidine  Methionine  Leucine  Lysine  Yeast Nitrogen Base + Tyrosine  Dextrose | 100  10  10  10  10  100  20gm |
| **YNB URA^-^  DEXTROSE + 750mM Salt**  **(False-positive)** | Dropout media  Histidine  Methionine  Leucine  Lysine  Yeast Nitrogen Base + Tyrosine  Dextrose  Sodium Chloride | 100  10  10  10  10  100  20gm  750 mM |
| **YNB URA^-^ Gal/Raff**  **(False-negative)** | Dropout media  Histidine  Methionine  Leucine  Lysine  Yeast Nitrogen Base + Tyrosine  Galactose  Raffinose | 100  10  10  10  10  100  20gm  0.5gm |
| **YNB URA^-^ Gal/Raff + Salt**  **(Experimental)** | Dropout media  Histidine  Methionine  Leucine  Lysine  Yeast Nitrogen Base + Tyrosine  Galactose  Raffinose  Sodium chloride | 100  10  10  10  10  100  20gm  0.5  750 mM |

* = pH adjusted to 7.2
